# Fiber intake and inflammation in type 1 diabetes

**DOI:** 10.1186/1758-5996-6-66

**Published:** 2014-05-29

**Authors:** Fernanda S R Bernaud, Mileni Vanti Beretta, Cigléa do Nascimento, Fabrícia Escobar, Jorge L Gross, Mirela J Azevedo, Ticiana C Rodrigues

**Affiliations:** 1 Departament of Internal Medicine, Universidade Federal do Rio Grande do Sul, Rua Ramiro Barcelos 2350, Prédio 12, 4° andar, 90035-003 Porto Alegre, RS, Brazil; 2 Division of Endocrinology of Hospital de Clínicas de Porto Alegre, Universidade Federal do Rio Grande do Sul, Rua Ramiro Barcelos 2350, Prédio 12, 4° andar, 90035-003 Porto Alegre, RS, Brazil

**Keywords:** Type 1 diabetes, Fiber intake, Inflammation

## Abstract

**Background:**

Higher intake of dietary fiber is associated with lower risk of coronary heart disease, the leading cause of mortality among people with type 1 diabetes. The protective effect includes the anti-inflammatory properties of some foods. Population-based studies have shown an inverse association between some nutritional habits and high sensitive -C-reactive protein (hs-CRP). This study aimed to ascertain the association between fiber intake and hs-CPR levels in patients with type 1 diabetes.

**Methods:**

This cross-sectional study was conducted with 106 outpatients with type 1 diabetes; age 40 ± 11 years; diabetes duration of 18 ± 8.8 years. Dietary intake was evaluated by 3-day weighed-diet records. Patients were categorized in 2 groups, according to fiber intake (>20 g/day and <20 g/day).

**Results:**

The group with fiber intake > 20 g/day had lower hs-CRP levels [median (25^th-^75^th^) 0.7 mg/dl (0.4-2.4) vs. 1.9 mg/dl (1.0-4.4); P = 0.002], than the other group. Controlled for HbA1c and energy intake, an inverse relation was observed between hs-CRP levels and total fiber [ß = − 0.030 (SE: 0.0120), P = 0.02], soluble fiber [ß = − 0.078 (SE: 0.0421), P = 0.06] and insoluble fiber [ß = − 0.039 (SE: 0.01761), P = 0.026]. Even, after additional adjustment fibers remained associated with lower hs-CRP levels. Total fibers were stratified in 4 groups: < 10 g/day, from 10 to < 20 g/day, from 20 to 30 g/day and > 30 g/day. Compared to the group who ingested < 10 g/day of total fiber (referent group), the group who consumed > 30 g/d had significantly lower hs-CRP levels [−2.45 mg/L, P = 0.012] independent of the HbA1c values.

**Conclusions:**

The present study suggests that an increased consumption of dietary fiber > 30 g/day may play a role in reducing inflammation in individuals with type 1 diabetes.

## Background

The consumption of dietary fiber, a group of polysaccharides and lignins of plant origin, has been associated with a lower risk of coronary heart disease (CHD) [1,2], the leading cause of mortality among people with type 1 diabetes (T1D) [3]. Epidemiologic studies have consistently shown that greater fruits and vegetables intakes are associated with a lower risk of incident cardiovascular events [4,5]. Individuals that consumed more than 5 fruits and vegetables servings/day had a 26% reduction in risk of stroke [6] and a 17% reduction in risk of coronary heart disease [7] compared with those consuming less than 3 servings/day. A prospective cohort study with 71,706 Swedish participants showed that fruit and vegetables consumption < 5 servings/day is associated with progressively shorter survival and higher mortality rates. They performed a dose–response analysis to evaluate 10th survival percentile differences (PDs) by using Laplace regression and estimated HRs by using Cox regression. Those who never consumed fruit and vegetables lived 3 years shorter (PD: −37 months [95% CI −58, −16 mo]) and had a 53% higher mortality rate (HR 1.53 [95% CI 1.19, − 1.99]) than did those who consumed 5 servings fruit and vegetables/day. Regarding fruit and vegetables separately, the study showed that those who never consumed fruit lived 19 months shorter (PD: −19 months [95% CI −29, −10 mo]) than did those who 1 fruit /day. Participants who consumed 3 vegetables/day lived 32 months longer than did those who never consumed vegetables (PD: 32 mo [95% CI 13, 51 mo]) [8]. Potential mechanisms for the protective effect of this class of food include its anti-inflammatory properties. Some population-based studies have shown an inverse association between nutrition and high sensitive -C-reactive protein (hs-CRP), a marker of systemic inflammation [9-11]. The high flavonoids fruit and vegetables diet increased endothelium-dependent microvascular reactivity (P = 0.017) with +2 portions/day (at 6 week over habitual intake) and reduced hs-CRP (P = 0.001) [12].

Particularly, a prudent dietary pattern characterized by high intakes of vegetables, fruit, and whole grains has shown to be related to lower concentrations of circulating CRP [13,14].

In type 2 diabetes (T2D) individuals, the intake of soluble fibers, mostly from whole-grain foods and fruits, had a protective role for the presence of MS [15]. Further, among women with T2D, intakes of whole grains, bran and cereal fiber were associated with lower levels of CRP [16].

It’s also well known that patients with diabetes have a chronic, low-grade inflammatory status [17], and it has been speculated that this low-grade systemic inflammation might be implicated in the pathogenesis of not only insulin resistance and the MS [18], but also of microvascular complications [19,20] and cardiovascular disease [19-21] in T1D.

Few studies have addressed the role of nutrition in individuals with T1D [22-24]. In men with T1D, increased saturated fat intake and lower intake of cereal fiber predicted a higher waist circumference (WC), whereas a lower polyunsaturated fat intake and a lower glycemic index of the diet determined lower WC [22].

Therefore, the main aims of this study were to describe the nutritional profile of a sample of subjects with T1D, stratified by fiber intake status, and to ascertain whether an association between hs-CRP levels and fiber intake exists.

## Methods

### Subjects

This cross-sectional study was conducted on a sample of patients with type 1 diabetes recruited consecutively from the outpatient endocrinology clinic of Hospital de Clínicas de Porto Alegre, a large teaching hospital in Porto Alegre, Brazil. Type 1 diabetes was defined as onset before 40 years of age, presence of ketonuria or ketonemia at the time of diagnosis, and dependence on insulin therapy to sustain life. Patients were selected on the basis of the following criteria: no dietary counseling by a registered dietitian during the 6 months preceding study enrollment, age >18 years, and duration of diabetes >5 years. Patients with renal failure, symptomatic heart failure (NYHA class III or IV), acute cardiovascular events in the preceding 6 months (stroke, myocardial infarction, or acute pulmonary edema), or inability to complete weighed dietary records were excluded from the sample. All current medications were continued, except for statins. Patients with hs-CRP levels ≥10 mg/L as measured by a high-sensitivity assay were also excluded, due to the possibility of other inflammatory conditions.

The recruitment process occurred from January 2011 to December 2011, and all participants provided written informed consent for participation. The study was conducted according to the guidelines laid down in the Declaration of Helsinki and the study protocol was approved by the Hospital de Clínicas de Porto Alegre research ethics committee.

### Clinical evaluation

All patients were questioned by a physician as to past medical history, current lifestyle, demographic data, and current medications. Subjects self-identified as white or nonwhite (mixed or black) and were classified as current smokers or noncurrent smokers. Current alcohol intake was categorized simply as present or absent depending on whether participants consumed any type of alcoholic beverage. The frequency of exercise, according to activities carried out during a typical day, was graded into four levels on the basis of a standardized questionnaire as described previously [25]. Level 1 was considered indicative of a sedentary lifestyle.

Sitting blood pressure was measured twice after a 10-min rest, in the left arm, using a digital sphygmomanometer (Omron® HEM-705 CP). Hypertension was defined as blood pressure ≥140/90 mmHg on two separate occasions or use of antihypertensive drugs [26]. All patients underwent a full physical examination.

Regarding nephropathy, patients were classified according to the results of a random spot urine sample or 24-h timed urine collection (at least two samples obtained 6 months apart). Patients were considered normoalbuminuric when the urinary albumin excretion rate (UAE) was < 17 mg/L or < 20 μg/min; microalbuminuric when the UAE was 17–174 mg/L or 20–199 μg/min; and macroalbuminuric when the UAE was > 176 mg/L or > 199 μg/min on at least two occasions in a 6-month period [27].

All patients were assessed for diabetic retinopathy (DR) by an ophthalmologist. DR was detected or evaluated by dilated direct and indirect ophthalmoscopy, and the severity was classified using the Global Diabetic Retinopathy Group scale [28], as ‘absence of DR’, ‘mild non-proliferative DR’ (NPDR), ‘moderate NPDR’, ‘severe DR’, or ‘proliferative DR’ (PDR). For purposes of analysis, patients were allocated into two groups (absence of DR or presence of DR regardless of severity).

The insulin resistance was calculated based on equation glucose disposal rate (eGDR) published previously [29].

### Nutritional evaluation

Body weight (to the nearest 100 g) and height (to the nearest 0.1 cm) were measured, with subjects barefoot and wearing lightweight clothing, using a column scale with height rod (Filizola®, Filizola Balanças Industriais S.A. Brazil). The body mass index (BMI) (kg/m^2^) was then calculated. Waist circumference was measured midway between the lower rib margin and the iliac crest, near the umbilicus, using flexible, non-stretch fiberglass tape.

Participants’ usual diets were assessed, by means of 3-day weighed dietary records (two nonconsecutive weekdays and one weekend day, with approximately 7 days apart between each weighted), one time, as previously standardized [30]. Subjects were issued commercially available scales (measurement range, 1–2000 g; CUORI®/CE-cuo-840, Italy) and measuring cups (25–250 mL; Marinex®, Brazil) and given detailed explanations and demonstrations of their use. Subjects were considered adherent when the ratio of protein intake estimated by weighed dietary records to protein intake estimated by nitrogen output (protein intake-weighed dietary records/protein intake-nitrogen ratio) was between 0.79 and 1.26 [31]. Completeness of urine collection was confirmed by 24-h urinary creatinine measurements.

The nutrient content of dietary records was analyzed using the Nutribase 2007 Clinical Nutritional Manager software version 7.14 (Cybersoft, Phoenix, AZ., USA) and updates [32]. Data were collected from January 2011 to December 2011. Data intakes from nutrients were expressed as a percentage of total daily energy (%), in crude amounts (g.day^–1^), and g/kg weight. Nutrient data on frequently consumed foods were updated if necessary and/or supplemented with data obtained from local manufacturers of specific industrialized foods.

The total, soluble and insoluble fiber content was estimated according to data provided in the CRC Handbook of Dietary Fiber in Human Nutrition [33]. To analyze the consumption of fiber according to origin, foods were classified as whole-grain (included both intact and pulverized forms), beans and legumes, fruits, tubers (potatoes, sweet potatoes, cassava and yams), and vegetables. We stratified the vegetables according to their carbohydrate content (%) of crude weight: vegetables from group A (5%) and from group B (10%). The following ingredients in the database were considered whole grains: whole wheat and whole wheat flour, whole oats and whole oat flour, whole cornmeal and whole corn flour, brown rice, whole rye and whole rye flour, whole barley and bulgur [34].

### Laboratory methods

The UAE rate was measured by immunoturbidimetry (MicroAlb Sera-Pak® immuno microalbuminuria; Bayer, Tarrytown, NY, on Cobas Mira Plus [Roche®]; mean intra-assay and interassay coefficients of variation, 4.5 and 7.6% respectively). HbA1c levels were measured by high-performance liquid chromatography (Merck-Hitachi 9100; Merck®, Darmstadt, Germany) (reference range 4.7–6.0%). Fasting plasma glucose was measured by the glucose-peroxidase colorimetric enzymatic method (Biodiagnostica®). Serum creatinine was measured by the Jaffe method, serum total cholesterol and triglycerides enzymatic colorimetric methods (ADVIA® 1800 AutoAnalyzer, Germany), and HDL cholesterol, by the homogeneous direct method (ADVIA® 1800 AutoAnalyzer, Germany). LDL cholesterol was calculated using the Friedewald formula [35]. hs-CRP was quantitated by turbidimetry (ADVIA® 1800 AutoAnalyzer, Germany), and fibrinogen was determined by the Clauss clotting method, which measures the rate of fibrinogen conversion to fibrin in a diluted sample under the influence of excess thrombin. Urinary urea was measured by an enzymatic ultraviolet assay (ADVIA® 1800 AutoAnalyzer, Germany).

### Statistical analysis

Data are presented as mean ± SD, frequency (%) or median (IQR). Baseline characteristics were compared by fiber intake status using Student’s *t* test, the Mann–Whitney *U* test or the chi-square (χ^2^) test. Pearson correlation was used to describe the correlation between hs-CRP (log transformed) and other variables. Hotelling t-test was used to compare correlations among the variables.

To determine the relationship between hs-CRP levels and fiber intake, we constructed a generalized linear model (with gamma regression) with hs-CRP levels as the dependent variable and fiber intake, glycemic control, age, sex, insulin dose, smoking, and intake of other nutrients as predictor variables.

All analyses were carried out in SPSS 18.0 (Chicago, IL).

## Results

Of 137 consecutive eligible patients, six refused to participate in the study, 14 had hs-CRP levels above 10 mg/L, and eleven were excluded due to compliance issues (inability to complete dietary records). Thus, 106 patients were included. The laboratory and clinical characteristics of the excluded group were not different from the subjects included in the study. Mean age was 40 ± 11 years, and mean duration of diabetes was 18 ± 8.8 years. Overall, 52.8% of subjects (n = 56) were men, most subjects were white (85.9%, n = 91), and nearly all were noncurrent smokers (91.5%, n = 97).

Table 1 shows key clinical and laboratory characteristics of patients stratified by median total fiber intake. Subjects in the higher fiber intake group (>20 g/d) were more likely to be male (71.2%), were less frequently sedentary (34.6%), used lower insulin doses (0.6 ± 0.2 UI/kg vs. 0.8 ± 0.3 UI/kg, P = 0.012), and had lower hs-CRP levels (median [IQR], 1.9 mg/dl [1.0-4.4] vs. 0.7 mg/dl [0.4-2.4]; P = 0.002) than those in the low fiber intake (<20 g/d) group. There were no significant differences in proportion of white subjects, current smoking, alcohol intake, presence of hypertension, blood pressure levels, BMI, waist circumference or presence of microvascular complications. Plasma glucose, HbA1c, and total, LDL and HDL cholesterol levels were similar in the two groups.

**Table 1:** Clinical and laboratory profile of subjects with type 1 diabetes, stratified by median total fiber intake

	**< 20 g/d**	**≥ 20 g/d**	**P**
**n**	**54**	**52**	
Age (years)	39.1 ± 11.6	40.9 ± 10.5	0.426†
Caucasian (%)	92.6	78.8	0.117‡
Male (%)	35.2	71.2	<0.001‡
Diabetes duration (years)	18.2 ± 9.5	17.8 ± 8.1	0.378†
Current smoking (%)	13.0	3.8	0.092‡
Frequency of exercise: level 1* (%)	61.1	34.6	0.028‡
Current alcohol intake (%)	44.4	42.3	0.824‡
BMI (kg/m^2^)	24.3 ± 3.6	24.5 ± 3.4	0.549†
Waist circumference (cm)			
Female	78.9 ± 9.2	81.0 ± 8.6	0.448†
Male	84.7 ± 8.4	86.0 ± 9.6	0.607†
Insulin (UI/kg)	0.8 ± 0.3	0.6 ± 0.2	0.012†
Office systolic blood pressure (mmHg)	125.1 ± 15.0	124.6 ± 17.6	0.867†
Office diastolic blood pressure (mmHg)	75.4 ± 11.7	74.3 ± 9.6	0.610†
Hypertension (%)	46.3	32.7	0.152‡
Presence of nephropathy (%)	16.7	11.5	0.449‡
Presence of diabetic retinopathy (%)	44.4	32.7	0.214‡
Fasting plasma glucose (mg/dl)	197.7 ± 132.0	200.2 ± 109.7	0.915†
HbA1c test (%)	9.1 ± 2.1	9.0 ± 1.9	0.752†
Total cholesterol (mg/dl)	190.4 ± 32.5	185.1 ± 36.7	0.434†
HDL cholesterol (mg/dl)			
Female	66.1 ± 14.9	69.1 ± 15.5	0.542†
Male	50.3 ± 10.2	53.4 ± 13.7	0.354†
LDL cholesterol (mg/dl)	112.0 ± 26.3	111.7 ± 38.6	0.959†
Triglycerides (mg/dl)	82.0 (59.8-114.2)	79.5 (57.8-114.8)	0.799†
hs-CRP (mg/L)	1.9 (1.0-4.4)	0.7 (0.4-2.4)	0.002†
Fibrinogen (mg/dl)	361.0 (311.5-448.0)	349.0 (271.0-410.0)	0.062†
UAEr (mg/24-h)	7.9 (0.0-21.9)	6.0 (0.0-12.0)	0.604†
Serum creatinine (mg/dl)	0.9 ± 0.4	0.9 ± 0.3	0.721†
eGDR (mg. kg^– 1^. min^– 1^)	7.76 ± 2.04	7.58 ± 2.59	0.700†

Data expressed as means ± SD, median (IQR), or n (%). *Level 1 = sedentary.

hs-CRP, high-sensitivity C-reative protein; UAE, urinary albumin excretion rate; eGDR equation glucose disposal rate. † Student’s *t* test; ‡ chi-square test.

Regarding daily nutrient intake, the group who consumed >20 g/d of fiber exhibited significantly higher total energy intake (kcal/d and kcal/weight) and higher carbohydrate and protein intake (crude and g/kg weight) than subjects who consumed up to 20 g/d fiber. There was a borderline difference in total energy intake from saturated fatty acids, and no differences in the percentage of total energy from protein, carbohydrates and fat (% energy). Individuals who ingested <20 g fiber/d consumed more saturated fat than those with higher fiber intake (10.4 ± 2.7% vs. 9.3 ± 3.5%, P = 0.059). There were no differences related to fiber intake status in total energy from saturated and monounsaturated fatty acids, even when both fatty acid types were regarded as a single category.

The mean daily intake of nutrients and total fibers from certain foods, stratified by median total fiber intake, is described in Table 2.

**Table 2:** Daily intake of nutrients among subjects with type 1 diabetes, stratified by median total fiber intake

	**< 20 g/d**	**≥ 20 g/d**	**P**
**n**	**54**	**52**	
Energy (kcal/day)	1842.1 ± 558.7	2376.4 ± 628.7	< 0.001†
Energy (kcal/weight)/day	28.6 ± 8.0	33.0 ± 8.4	0.006†
Carbohydrates			
Crude intake (g)	220.0 ± 66.1	302.1 ± 88.0	< 0.001†
Weight (g/kg)	3.4 ± 1.0	4.2 ± 1.3	< 0.001†
Energy (%)	48.4 ± 8.0	51.4 ± 8.6	0.074†
Protein			
Crude intake (g)	82.6 ± 31.5	108.3 ± 35.3	< 0.001†
Weight (g/kg)	1.3 ± 0.5	1.5 ± 0.4	0.007†
Energy (%)	18.0 ± 3.7	18.3 ± 3.4	0.616†
Total fat			
Crude intake (g)	70.0 ± 31.6	82.4 ± 35.3	0.011†
Weight (g/kg)	1.1 ± 0.4	1.1 ± 0.5	0.394†
Energy (%)	33.6 ± 8.8	30.7 ± 9.6	0.110†
Saturated fatty acid			
Crude intake (g)	22.1 ± 10.4	24.5 ± 10.8	0.247†
Weight (g/kg)	0.3 ± 0.1	0.3 ± 0.1	0.952†
Energy (%)	10.4 ± 2.7	9.3 ± 3.5	0.059†
Monounsaturated fatty acid			
Crude intake (g)	24.6 ± 11.6	28.4 ± 12.7	0.107†
Weight (g/kg)	0.4 ± 0.2	0.4 ± 0.2	0.530†
Energy (%)	11.7 ± 3.5	10.6 ± 3.4	0.098†
Polyunsaturated fatty acid			
Crude intake (g)	11.6 (8.3-19.9)	18.4 (10.6-26.0)	0.026§
Weight (g/kg)	0.2 (0.1-0.3)	0.2 (0.1-0.3)	0.148§
Energy (%)	6.0 (4.2-10.0)	7.1 (4.6-0.4)	0.733§
*Trans* fatty acid – crude intake (g)	0.1 (0.0-0.3)	0.1 (0.0-0.4)	0.069§
Cholesterol (mg)	239.9 ± 148.4	239.0 ± 116.3	0.972†
Total fiber (g)			
Crude intake (g)	14.6 ± 3.5	27.2 ± 6.8	< 0.001†
Weight (g/kg)	0.2 ± 0.1	0.4 ± 0.1	< 0.001†
Soluble fiber			
Crude intake (g)	4.3 ± 1.3	7.8 ± 2.1	< 0.001†
Weight (g/kg)	0.1 ± 0.0	0.1 ± 0.0	< 0.001†
Insoluble			
Crude intake (g)	10.5 ± 2.8	19.4 ± 5.1	< 0.001†
Weight (g/kg)	0.2 ± 0.1	0.3 ± 0.1	< 0.001†
Fibers from fruits			
Total fiber (g)	1.4 (0.2-2.7)	3.0 (1.4-4.9)	0.005§
Fibers from vegetables (A + B)			
Total fiber (g)	1.1 (0.5-2.5)	2.8 (1.5-4.0)	0.001§
Fibers from Tuberous			
Total fiber (g)	0.4 (0.0-0.76)	0.2 (0.0-0.8)	0.062§
Fibers from whole-grain foods			
Total fiber (g)	0.4 (0.0-3.8)	1.1 (0.0-4.5)	0.104§
Fibers from legumes and beans			
Total fiber (g)	2.5 (0.1-6.7)	8.4 (5.5-12.7)	< 0.001§

Data expressed as means ± SD or median (IQR).

† Student’s *t* test; § Mann–Whitney *U* test.

Subjects with a total intake of >20 g fiber/day consumed more fiber from fruit, A and B vegetables, legumes, and beans than subjects in the low-fiber group.

To determine the relationship between fiber intake and hs-CRP levels, additional analyses were performed. First, to understand other variables possibly involved with inflammatory status, correlations between hs-CRP levels (log-transformed) and others variable were calculated. The hs-CRP level correlated with HbA1c (r = 0.29, P = 0.002), eGDR (r = −0.286, P = 0.003), total energy intake (kcal/day and kcal/weight) (r = −0.26, P = 0.008; r = −0.31, P = 0.001, respectively), protein intake (crude and g/kg) (r = − 0.34, P = 0.001; r = −0.39, P <0.001), and carbohydrate intake (crude and g/kg) (r = −0.26, P = 0.007; r = −0.28, P = 0.004), as well as with total dietary fiber intake (crude and g/kg) (r = −0.30, P = 0.002; r = −0.33, P < 0.001), soluble fiber intake (crude and g/kg) (r = −0.24, P = 0.012; r = −0.27, P = 0.005) and insoluble fiber intake (crude and g/kg) (r = −0.30, P = 0.002; r = −0.33, P = 0.001). In this sample, fatty acid intake did not correlate with hs-CRP levels.

Hotelling t-test was used to compare correlations among the variables, we observed that there is an effect of the protein on the correlation between hs-CRP and fiber intake, as well there is an effect of the fiber on the correlation between the protein intake and hs-CRP. The partial squared correlation coefficients for fiber, protein and carbohydrates in models adjusting for age, sex, HbA1c, smoking and insulin dose was 0.26, 0.29 and 0.30 respectively for carbohydrates, proteins and fibers.

In a regression linear model with the hs-CRP as dependent variable and age, sex, HbA1c, smoking, insulin dose, energy (kcal/day) and fiber intake as independent variables, the fiber remained associated with hs-CRP levels independent of the adjustment of the others variables. However, when the fiber was replaced by protein or carbohydrates intake, with the same adjusts, protein and carbohydrates were not associated with the hs-CRP.

When, the stratification for fiber (below and above of the median), adjusted for age, sex, HbA1c, smoking and insulin dose was performed in multiple regression model. The fiber > 20 g/day was associated with the hs-CRP levels (beta = − 0.281, p = 0.005).

The regression coefficients of dietary total fiber, soluble fiber and insoluble fiber for predicting hs-CRP levels from a generalized linear model are shown in Table 3. The results of analyses unadjusted and adjusted for covariates are presented. After controlling for HbA1c and energy intake, a significant inverse correlation was observed between hs-CRP levels and total, soluble and insoluble fiber intake. After adjusting for HbA1c and protein or carbohydrate intake, total, soluble and insoluble fiber intake remained inversely associated with lower hs-CRP levels. Notably, HbA1c also remained associated with hs-CRP levels in all models constructed, including sex, smoking and physical activity.

**Table 3:** Regression coefficients (beta, ß) of dietary total, soluble and insoluble fiber for prediction of hs-CRP levels according to a generalized linear model

	**Unadjusted**		**Adjusted**^**1**^		**Adjusted**^**2**^		**Adjusted**^**3**^	
	**ß (SE)**	**P**	**ß (SE)**	**P**	**ß (SE)**	**P**	**ß (SE)**	**P**
Total fiber (g/day)	−0.039 (0.0118)	0.001	−0.030 (0.0120)	0.02	−0.037 (0.0140)	0.007	−0.042 (0.0134)	0.002
Soluble fiber (g/day)	−0.106 (0.0417)	0.011	−0.078 (0.0421)	0.06	−0.092 (0.0426)	0.03	−0.098 (0.0426)	0.022
Insoluble fiber (g/day)	- 0.053 (0.0160)	0.001	−0.039 (0.0176)	0.026	−0.050 (0.0194)	0.009	−0.057 (0.0184)	0.002

^1^Adjusted for HbA1c and energy intake (kcal/weight).

^2^Adjusted for HbA1c and weight (g/kg) of protein.

^3^Adjusted for HbA1c and weight (g/kg) of carbohydrate.

**Figure 1:**
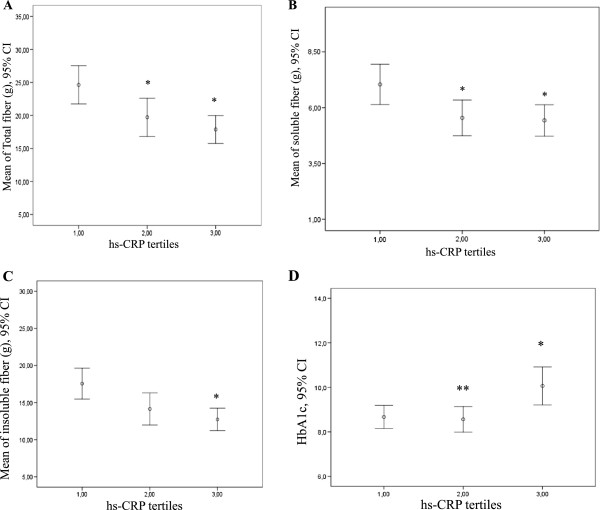
**Unadjusted mean total fiber intake (A), soluble fiber intake (B), insoluble fiber intake (C) and HbA1c% (D) by hs-CRP tertiles (mg/L) in 106 subjects with type 1 diabetes.** hs-CRP levels stratified into tertiles (first: 0.0–0.72 mg/L; second: 0.721–2.81 mg/L; third: 2.811–10 mg/L).* different from the first tertile and ** different from the third tertile with P < 0.001.

On the basis of these results, and to examine potential differences between the amount of fiber intake and HbA1c levels in relation to hs-CRP concentrations, we constructed models with total fiber intake stratified into four groups (<10 g/day, from 10 to < 20 g/day, 20–30 g/day and >30 g/day) and adjusted for HbA1c (stratified into tertiles). The regression coefficients of these variables for predicting hs-CRP from a generalized linear model are presented in Table 4. Compared to the group who ingested < 10 g/day total fiber (referent group), subjects who consumed >30 g/day had significantly lower hs-CRP levels (−2.45 mg/L, P = 0.012) regardless of HbA1c values. Furthermore, as compared with the lowest HbA1c tertile (the referent group), subjects in the highest tertile had higher hs-CRP levels (1.387 mg/L, P = 0.006) regardless of fiber intake.We also examined the associations between total, soluble and insoluble fiber intake and HbA1c with hs-CRP levels stratified into tertiles (0.0–0.72 mg/L, 0.721–2.81 mg/L and 2.811–10 mg/L), as illustrated in Figure 1. Subjects with hs-CRP levels in the second and last tertiles had significantly lower total fiber and soluble fiber intake (Figure 1-A and B) when compared with individuals in the first hs-CRP tertile (those with the lowest hs-CRP levels). No difference in intake was observed between the second and third tertiles. Subjects in the highest hs-CRP tertile consumed less insoluble fiber than subjects in the first tertile (Figure 1-C). We also observed that subjects with hs-CRP levels in the third tertile had higher HbA1c levels than those in the second and first hs-CRP tertiles (Figure 1-D). There was no difference in HbA1c levels between subjects in the second and first hs-CRP tertiles.

**Table 4:** Regression coefficients (beta, ß) of different amounts of dietary total fiber and HbA1c tertiles for prediction of hs-CRP levels according to a generalized linear model

	**ß (SE)**	**P**
Total fiber		
<10 g/day	-	-
10 - < 20 g/day	−0.507 (0.8407)	0.546
20 - 30 g/day	−0.986 (0.8562)	0.250
>30 g/day	−2.450 (0.9744)	0.012
HbA1c		
Tertile 1	-	-
Tertile 2	0.186 (0.4992)	0.709
Tertile 3	1.37 (0.5041)	0.006

SE, standard error. HbA1c tertiles: 6.3–8.0%, 8.01–9.3%, and > 9.3%.

## Discussion

The present study showed an inverse association between serum hs-CRP levels and intake of dietary fiber (total, soluble and insoluble fiber) in persons with T1D. Subjects who consumed higher amounts of dietary fiber had lower hs-CRP levels. After adjusting for possible confounders, this association persisted for all types of dietary fiber intake. Total dietary fiber intake in excess of 20 g/day was associated with even lower levels of hs-CRP. Notably, the level of total dietary fiber intake (>30 g/day) found to be associated with lower hs-CRP levels in this study was not different from that recommended for the general population and for persons with diabetes by the American Diabetes Association (14 g/1000 kcal) [36].

In the study sample, the median total dietary fiber intake was 20 g/day. The main sources of dietary fiber in the >20 g/day group were fruits, vegetables (A + B), legumes and beans. A prospective study evaluated the association between quantity and variety of fruit and vegetable intake and incidence of T2D [37]. Their results suggested that a greater combined fruit and vegetable intake was associated with a 21% lower risk of T2D (HR 0.79 [95% CI 0.62-1.00]) on comparison of extreme tertiles (highest tertile = 3.4 [2.9-4.4] servings/day and lowest tertile = 0.6 [0.3-0.9] servings/day), in adjusted analyses taking variety into account. When the authors compared extreme tertiles of fruit variety (6.9 ± 1.2 vs. 2.0 ± 1.0 items/week), vegetable (11.4 ± 1.5 vs. 5.5 ± 1.4) and combined fruit and vegetable intake (16.3 ± 8.0 vs. 8.0 ± 1.8), a greater variety in these intakes was associated with lower risks of T2D (30%, 22% and 39% respectively).

The protective role of foods rich in soluble fibers has been previously evaluated in T2D patients with MS [15]. Total fiber and soluble fiber intake from whole-grain foods and fruits were negatively associated with MS. The mean daily intake of total fiber (20.3 ± 7.8 g/day) of patients without MS in that study was similar to the median (IQR) (20.0 [14.9-25.5]) of daily total fiber intake in our study of T1D patients. Data from the EURODIAB Complications Study [22], a cross-sectional investigation of T1D patients, also reported a mean total fiber intake of 20.0 g/day and a percentage of energy intake from protein of 17.4%, both of which are similar to our findings. The total fat intake observed in the European study (percentage of energy from fat, 37.9%) was higher than that observed in our study, regardless of fiber intake status (33.6% in the < 20 g fiber/day group vs. 30.7% in the >20 g fiber/day group). This difference is explained by higher intake of monounsaturated and saturated fats. A similar finding was also reported in a U.S. population of persons with T1D [23], i.e. a higher percentage of energy from saturated fat as compared with that found in our sample (12.9% vs. 10.4%). Our patients had higher carbohydrate intake than that observed in both of the above-cited studies: percentage of energy from carbohydrates, 48.4% vs. 41.9% [22] and 44% [23]. These data demonstrate the lack of literature on fiber intake in T1D populations.

The association between high fruit and vegetable intake and a lower risk of mortality from ischemic heart disease (IHD) was demonstrated in the European Prospective Investigation into Cancer and Nutrition (EPIC)-Heart Study [38], where subjects who ate at least eight servings (80 g each) of fruits and vegetables per day had a 22% lower risk of fatal IHD compared with those who consumed fewer than three portions a day.

The role of inflammation in the etiology of several chronic diseases—perhaps the most notable being cardiovascular disease (CVD), diabetes and several types of cancer—is supported by epidemiological evidence [39-41]. CRP levels consistently predict positive associations with chronic conditions, including malignant disease [42-45].

Although several studies have examined associations between dietary fiber intake and serum hs-CRP levels [16,46-48] there are no available data in the literature about the effects of dietary fiber on systemic inflammation in populations with T1D. Our findings are consistent with those of previous observational and clinical intervention studies that investigated the relationship between dietary fiber intake and serum hs-CRP levels [16,46,48,49] in the general population. In our sample, subjects with T1D who consumed >30 g/day of total fiber had lower levels of hs-CRP (−2.450 mg/L) than those who ingested <10 g/day.

Ma et al. [49], using cross-sectional and longitudinal data from 524 healthy adult participants of the Seasonal Variation of Blood Cholesterol Levels Study (SEASONS), reported an inverse association between intake of total dietary fiber (separately for soluble and insoluble fiber) and hs-CRP concentrations. The coefficient for the cross-sectional effect of dietary total fiber was −0.01, and −0.02 for insoluble dietary fiber, adjusted for covariates. Our findings for the effect were somewhat higher: −0.042 for total fiber, −0.098 for soluble fiber, and −0.057 for insoluble fiber. When we stratified total fiber intake, this effect was even greater in the >30 g fiber/day group. This may be attributable to differences in the characteristics of the studied population, and the effect of fiber in persons with T1D may be greater than in non-diabetic individuals.

Using cross-sectional data from the general population, Ajani et al. reported that the odds ratio (OR) of elevated hs-CRP levels was 0.49 (95% CI: 0.37-0.65) for the highest quintile of total fiber intake (32 g/day) as compared with the lowest quintile (5.1 g/day) [46].

In a multiethnic cohort of early-stage breast cancer survivors, Villaseñor et al. [47] investigated the relationship between intake of total, soluble, and insoluble dietary fiber and hs-CRP levels. Inverse associations were found between total fiber intake (ß = −0.029; 95% CI −0.049, −0.008; P = 0.006), insoluble fiber intake (ß = −0.039; 95% CI −0.064, −0.013; P = 0.003) and hs-CRP levels. When only hs-CRP levels ≥3 mg/L and tertiles of total, soluble and insoluble dietary fiber intake were taken into account, the association held only for the highest tertile of insoluble fiber intake (mean intake, 15.5 ± 3.4 g/day).

de Mello et al. observed that, after 3 months of a dietary intervention consisting of an experimental diet high in fatty fish, bilberries and wholegrain products (Healthy Diet) or a whole-grain-enriched (WGED) diet, subjects with impaired glucose metabolism and features of MS had lower hs-CRP levels than controls assigned to a low-fiber diet. The authors also observed that both intervention diets (Healthy and WGED) were associated with a reduction in 2-hour plasma glucose levels as compared with the control diet (mean ± SD: 120.7 ± 30.6 mg/dL vs. 109.9 ± 30.6 mg/dL and 118.9 ± 28.8 mg/dL vs. 109.9 ± 34.2 mg/dL respectively) [50]. HbA1c levels were not reported.

However, not all randomized clinical trials support the beneficial role of fiber, at least from wholegrains, in inflammation or cardiovascular risk factors. The Women’s Health Initiative Dietary Modification Trial [51], a randomized controlled trial including more than 48,000 postmenopausal women and followed for 8 year, showed an increased risk of 1.26 [95% CI 1.03 - 1.54] for CVD in those women with cardiovascular disease CVD at baseline and allocated to the intervention arm. The intervention consisted in a low-fat diet with more than 6 servings/day of wholegrains, and 5 servings/day of fruits and vegetables. Lefevre et al. concluded that epidemiological studies provide reasonable support for an association between diets high in whole grains and lower CRP concentrations. After adjusting for other dietary factors, each serving of whole grains is estimated to reduce CRP concentrations by approximately 7%. Nonetheless, interventional studies do not demonstrate a clear effect of increased whole-grain consumption on CRP or other markers of inflammation [52].

The cross-sectional nature of this study precludes evaluation of causality due to the uncertain temporality of associations. Nevertheless, this design allowed us to assess the usual diet of T1D patients. The high average HbA1c levels found in this sample are also a potential limitation. However, the cohort enrolled in this study was composed of unselected adults with T1D treated routinely at an outpatient endocrinology clinic and can thus be considered representative of real patients outside a clinical trial setting, as already observed in the multicenter study conducted in different regions of Brazil [53].

In conclusion, the results of the current study show that a higher consumption of dietary fiber is associated with lower hs-CRP levels in persons with T1D. High dietary fiber intake (>30 g/day) may play a role in reducing inflammation in this population. Randomized controlled trials of high- and low-fiber diets are required to support public health recommendations.

## Abbreviations

T1D: Type 1 diabetes; CRP: C-reactive protein; T2D: Type 2 diabetes; WC: Waist circumference; HS: High sensitive; UAE: Urinary albumin excretion; DR: Diabetic retinopathy; NPDR: Non-proliferative diabetic retinopathy; BMI: Body mass index; IHD: Ischaemic heart disease; CVD: Cardiovascular disease; MS: Metabolic syndrome.

## Competing interests

The authors declare that they have no competing interests.

## Authors’ contributions

FRB researched data, and wrote the manuscript, MVB, CN and FE researched data, MJA reviewed the manuscript, JLG contributed to the discussion and TCR researched data, and wrote the manuscript. All authors read and approved the final manuscript.
